# Spatiogenetic characteristics of beech stands with different degrees of autochthony

**DOI:** 10.1186/1472-6785-5-8

**Published:** 2005-12-06

**Authors:** Hans-Rolf Gregorius, Dierk Kownatzki

**Affiliations:** 1 lnstitut für Forstgenetik und Forstpflanzenzüchtung, Universität Göttingen, Büsgenweg 2, 37077 Göttingen, Germany

## Abstract

**Background:**

Autochthony in forest tree stands is characterized by a number of criteria, among which the range over which stands act as a population has been suggested to play a central role. Therefore, measures are needed for the delineation of populations or the detection of subpopulation structure. It is argued here that methods of population delineation must be based on the combined consideration of spatial distances and genetic differences between adult individuals. Conventional approaches and a set of newly developed methods are applied to seven isozyme loci in four beech stands which are distinguished by different types of forest management based on natural regeneration.

**Results:**

Permutation analyses show that correlations between spatial distances and genetic differences vary only little in the studied beech stands. In view of the popularity of this and related descriptors of spatiogenetic covariation, this result came as a surprise. The newly developed methods lead to a different conclusion. Significant spatiogenetic structure is indicated in all stands when considering the mean and variance of spatiogenetic separation, where separation is measured by the smallest spatiogenetic difference of an individual from all others. Spatiogenetic difference is measured here by a combination of the spatial distances and genetic differences between individuals. This descriptor indicates the existence of spatiogenetic clusters in the beech stands. In order to arrive at an explicit representation of cluster structure as a representation of subpopulation structure, two types of cluster structure (primary and *α*-isolated) are distinguished, both of which reflect desirable characteristics of subpopulation structure. Particularly in the *α*-isolated structure, the proportion of individuals organized in clusters, the effective size, and the effective number of clusters clearly distinguish and consistently rank the four stands with respect to their types of forest management and the associated criteria of autochthony.

**Conclusion:**

The surprisingly high correspondence between our descriptors of spatiogenetic structure and forest management types confirms the appropriateness of the applied measure of cluster isolation and of the criterion for the choice of the level *α *of cluster isolation. The two types of cluster structure and their characteristic descriptors are thus suggested to be promising tools for the detection of subpopulation structure. To include the effects of long-distance gene flow, the presented methods can be extended as outlined to larger spatial scales in order to detect higher order population structure.

## Background

Autochthony is generally considered for conspecific collections of individuals existing at a specified location. Basically, autochthony is judged by the time over which the ancestry of the collection has existed as a population at this location. In a recent paper by Kleinschmit et al. [1] the close relationship between the autochthony of a stand and its state as a population was reasoned in some detail. This relationship is due to the local, temporal and genealogical continuity as well as the resulting adaptational specificity associated with the notion of autochthony. Particularly the potential of reaching high degrees of adaptedness under long term regular conditions, as are imputed to autochthonous stands, requires a proper balance between internal reproductive coherence and external reproductive isolation over generations for its realization. These are in fact the generally agreed upon characteristics of (Mendelian) populations, and they imply the delineation of a spatial and environmental range within which the interaction between selection and gene flow or mating system characteristics presents a population as a unit of adaptation and reproduction. Hence the conclusion of Kleinschmit et al. [1] that the autochthony of a stand involves as an essential determinant the range, over which it acts as a population.

Almost all information on reproductive coherence in plants species is due to gene flow studies between more or less artificially delineated units or stands [2-4] and (less frequently) to mating relations (for tree species see e.g. [5-7]) within such stands. The significance of the interplay between gene flow and adaptation, however, is usually a matter of the discussion sections in research reports. Even more so the same seems to hold true in the reverse direction, where experimental studies are focused on the detection of adaptational processes, and where gene flow is not explicitly considered as to its effects on the range of the habitat to which adaptation has to take place. Even the plethora of models treating joint effects of gene flow and selection resort to pre-defined populations or ranges, so that they cannot be applied to the (indirect) estimation of population ranges or to the detection of population subdivisions (for a review on these topics see e.g. [4]).

To overcome the problems of inference resulting from separate consideration of effects of gene flow and adaptation as well as from artificial delineation of stands or units in experimental studies, the integrated approach to population delineation suggested by Kleinschmit et al. [1] will be further developed in this paper. Rather than estimating rates of gene flow and selection, the approach focuses on the outcome of the joint action of both processes as it shows in the spatial distribution of genetic information (i.e. *spatiogenetic structure*). This is based on the reasoning that in order for a collection of individuals to constitute a population

1. matings within the collection should be distinctly more frequent than matings with individuals from outside the collection,

2. offspring with one parent from the collection and the other from outside the collection should be less likely to survive to adulthood or to stay within the collection than offspring with both parents from the collection (this can be expected to be even more true if both parents originate from outside the collection).

At least in plants, collections of individuals are primarily identified by their spatial distribution, and parent-offspring relations in combination with the local adaptational processes establish specific genetic structures. Hence, fulfillment of the two conditions should indeed imply distinct spatiogenetic structures among adults after at least two generations. Consideration of adult individuals is required, since earlier stages such as the seed stage may appropriately reflect effects of the mating system but do not sufficiently account for selection processes. Adult stages summarize the outcome of the joint action of mating and selection. The problem of assessing autochthony thus presents itself largely as one of finding natural substructures in stands as indicators of combined adaptational and reproductive separation at a small or medium scale. Reconstruction of population history is not of primary relevance in this concern. It is rather the presently observable outcome of the past action of population characteristics that matters. This is approached in two steps: (a) by finding associations between genetic differences and spatial distances, and (b) by identifying and characterizing groups of individuals which form spatiogenetic clusters. Both of these aspects have to be analyzed independently as long as implications of one by the other are not obvious. While methods of type (a) can be selected from a large supply (for a review see e.g. [8]), methods of type (b) do not yet seem to have attracted much interest [9]. On the other hand, these methods are indispensable in order to not just indicate the existence but to concretely identify the groups of individuals, which show tendencies for the formation of subpopulations or populations. Moreover, the large scale, on which associations are to be determined may show no significance despite the existence of smaller scale clustering.

To start with, we chose beech (*Fagus sylvatica *L.) as a dominant forest tree species in central Europe. Beech has been studied intensively for its genetic variation within and between stands with the general result that it appears to be less differentiated among stands than other comparable tree species (see e.g. [10-12]). As potential explanations of this observation, gene flow and post-glacial re-migration were discussed in combination with the species's dispersal mechanisms and ecological state as a climax species (for a review see [13]). Small scale studies showed that pollen dispersal detectable from paternal analyses might be limited (though to quite variable degrees, [7,13]), and local selection is likely to act, as was chiefly concluded from pairwise sampling studies [14,15].

The apparent absence of distinct, large scale genetic differentiation, the restricted capacity of seed dispersal, and the possibility of locally acting selection pressures may be felt to be contradictory and therefore gave reason for the present study to concentrate on an analysis of medium scale structures in spatially more or less continuous stands. By this it is intended to check for the existence of spatiogenetic structures that indicate tendencies of forming subpopulations that cannot be distinguished in neither large nor small scale studies. For this purpose a number of beech stands is selected that differ in their autochthony characteristics. These characteristics conform with the most commonly applied criteria of autochthony. The criteria relevant for the present study are compiled and briefly explained in an Appendix. The gene markers available in the present study are isoenzymes, which have been argued to be adaptive in some contexts and selectively neutral in others. Together with the possibility of being stochastically associated with directly selected genetic traits, these gene markers can thus be expected to be involved in the above-mentioned conditions of population delineation.

Four beech stands, three of them are located in Hessen, and one in Westfalia, have been selected because they represent different types of forest management. All trees of a trial had been spatially mapped and genetically characterized at seven polymorphic isozyme gene loci (PGM, IDH, SDH, AP, 6PGDH, MNR, MDH) in earlier studies [16-18]. Before compilation the genetic data were adjusted to a standardized nomenclature of genetic variants. The stand characteristics are summarized in Table 2.

**Table 1:** Description of three commonly used measures of genetic difference between individuals. Each diploid individual is characterized by two *individual genes *at each gene locus, which are either identical in their *gene state *(= homozygous; e. g. *A*_1_*A*_1_) or different in their *gene state *(= heterozygous; e. g. *A*_1 _*A*_2_). Measurements of genetic difference between two individuals can therefore be based on either differences in number of *gene states *(*d*_*J*_) or on number of *individual genes *differing in gene state (*d*_0_, *d*_*ur*_) at a specified number of gene loci.

*formal representation*	*verbal description*
d0=∑kmax{G10.k, G01.k}2⋅l MathType@MTEF@5@5@+=feaafiart1ev1aaatCvAUfKttLearuWrP9MDH5MBPbIqV92AaeXatLxBI9gBaebbnrfifHhDYfgasaacH8akY=wiFfYdH8Gipec8Eeeu0xXdbba9frFj0=OqFfea0dXdd9vqai=hGuQ8kuc9pgc9s8qqaq=dirpe0xb9q8qiLsFr0=vr0=vr0dc8meaabaqaciGacaGaaeqabaqabeGadaaakeaacqWGKbazdaWgaaWcbaGaeGimaadabeaakiabg2da9maalaaabaWaaabeaeaatCvAUfeBSjuyZL2yd9gzLbvyNv2CaeHbwvMCKfMBHbaceiGaa8xBaiaa=fgacaWF4baaleaacqWGRbWAaeqaniabggHiLdGcdaGadaqaaiaa=DeadaWgaaWcbaGaeGymaeJaeGimaaJaeiOla4Iaem4AaSgabeaakiabcYcaSiabbccaGiabdEeahnaaBaaaleaacqaIWaamcqaIXaqmcqGGUaGlcqWGRbWAaeqaaaGccaGL7bGaayzFaaaabaGaeGOmaiJaeyyXICTaemiBaWgaaaaa@53DD@	*the minimum number of individual genes, the states of which have to be replaced in one individual in order to get the genotype of the other individual*
dJ=∑k(G10.k, G01.k)∑k(G11.k+G10.k+G01.k) MathType@MTEF@5@5@+=feaafiart1ev1aaatCvAUfKttLearuWrP9MDH5MBPbIqV92AaeXatLxBI9gBaebbnrfifHhDYfgasaacH8akY=wiFfYdH8Gipec8Eeeu0xXdbba9frFj0=OqFfea0dXdd9vqai=hGuQ8kuc9pgc9s8qqaq=dirpe0xb9q8qiLsFr0=vr0=vr0dc8meaabaqaciGacaGaaeqabaqabeGadaaakeaacqWGKbazdaWgaaWcbaGaemOsaOeabeaakiabg2da9maalaaabaWaaabeaeaadaqadaqaaiabdEeahnaaBaaaleaacqaIXaqmcqaIWaamcqGGUaGlcqWGRbWAaeqaaOGaeiilaWIaeeiiaaIaem4raC0aaSbaaSqaaiabicdaWiabigdaXiabc6caUiabdUgaRbqabaaakiaawIcacaGLPaaaaSqaaiabdUgaRbqab0GaeyyeIuoaaOqaamaaqababaWaaeWaaeaacqWGhbWrdaWgaaWcbaGaeGymaeJaeGymaeJaeiOla4Iaem4AaSgabeaakiabgUcaRiabdEeahnaaBaaaleaacqaIXaqmcqaIWaamcqGGUaGlcqWGRbWAaeqaaOGaey4kaSIaem4raC0aaSbaaSqaaiabicdaWiabigdaXiabc6caUiabdUgaRbqabaaakiaawIcacaGLPaaaaSqaaiabdUgaRbqab0GaeyyeIuoaaaaaaa@589B@	*the number of gene states present in only one of the two individuals among the total number of gene states present in both individuals*
dur=∑kGk4⋅l MathType@MTEF@5@5@+=feaafiart1ev1aaatCvAUfKttLearuWrP9MDH5MBPbIqV92AaeXatLxBI9gBaebbnrfifHhDYfgasaacH8akY=wiFfYdH8Gipec8Eeeu0xXdbba9frFj0=OqFfea0dXdd9vqai=hGuQ8kuc9pgc9s8qqaq=dirpe0xb9q8qiLsFr0=vr0=vr0dc8meaabaqaciGacaGaaeqabaqabeGadaaakeaacqWGKbazdaWgaaWcbaGaemyDauNaemOCaihabeaakiabg2da9maalaaabaWaaabeaeaacqWGhbWrdaWgaaWcbaGaem4AaSgabeaaaeaacqWGRbWAaeqaniabggHiLdaakeaacqaI0aancqGHflY1cqWGSbaBaaaaaa@3CAF@	*the expected relative degree of heterozygosity of the off- spring of two individuals*

*l *:= number of gene loci, *G*_10.*k *_:= number of gene states at the *k*-th locus present only in the first individual and absent in the second, G_01.*k *_:= number of gene states at the *k*-th locus present only in the second individual and absent in the first, *G*_11.*k *_:= number of gene states at the *k*-th locus present in both individuals, *G*_*k *_:= number of heteroallelic pairs of individual genes, one from each of the two individuals.

**Table 2:** Characteristics of the studied stands

stand	area [ha]	number	age	regeneration	management
Laubach A	0.5	132	156 ± 10	nr.	w. lth.
Laubach C	0.5	71	156 ± 10	nr.	h. lth.
Horn	1.5	164	126 ± 10 43 ± 10	nr. & pl. nr.	hth.
Karlshafen	1.5	142	150 ± 10	nr. & pl.	hth.

nr.=: natural regeneration; lth.=: low thinning (weak low thinning is reported to be similar to competitive self-thinning); w.=: weak; h.=: heavy; pl.=: planting; hth.=: high thinning; planting in the Horn stand comes from local origins and in the Karlshafen stand from unknown origin

## Results

### Spatiogenetic correlation and asymmetry

Table 3 shows that the observed spatiogenetic correlations are close to zero for all three measures of genetic difference and in all four stands. This indicates a type of structure that deviates strongly from a linear relationship between genetic difference and spatial distance and is likely to be the result of a more erratic assignment of genotypes to spatial positions. The type of structure is not extreme as can be taken from the non-significant *p*-values. However, the effective ranges of variation of the correlations obtained from 10.000 permutations are quite narrow, indicating that there was not much potential for deviation from the observed structural characteristics anyhow. Considering the sizable genetic diversity in the stands, larger effective ranges of variation could have been expected. This observation reveals spatiogenetic correlation as a probably not very sensitive descriptor of spatiogenetic structure at least under the spatial and genetic marginal conditions of the four studied stands.

**Table 3:** Observed correlations between spatial distance and genetic difference, and their effective ranges of potential variation for three measures of genetic difference

stand	*d*_0_		*d*_*J*_		*d*_*ur*_	
	*c*_*sg*_	*p*	*c*_*sg*_	*p*	*c*_*sg*_	*p*
Laubach A	**.0232 ** (-.0441; 0453)	.19	**.0120 ** (-.0395; .0407)	.31	**.0350 ** (-.0509; .0521)	.14
Laubach C	**.0202 ** (-.0602; .0619)	.28	**.0203 ** (-.0562; .0592)	.28	**.0177 ** (-.0655; .0677)	.32
Horn	**.0146 ** (-.0415; .0416)	.28	**.0098 ** (-.0340; .0356)	.31	-**.0153 ** (-.0515; .0523)	.31
Karlshafen	**.0077 ** (-.0422; .0428)	.38	**.0132 ** (-.0370; .0377)	.28	-**.0182 ** (-.0507; .0520)	.72

*c*_*sg *_:= spatiogenetic correlation coefficient, *p *:= proportion of correlation coefficients among 10.000 permutations exceeding *c*_*sg*_, second line in parentheses:= lower and upper 0.05-quantile specifying the effective range of variation of spatiogenetic correlation obtained for 10.000 permutations

For average spatiogenetic asymmetry the situation is even less discriminative between stands (see Table 4). In fact, there is almost no difference between the stands for all measures of genetic difference, and the effective ranges of variation across permutations consequently overlap substantially and are very narrow. Moreover, the average asymmetries are of intermediate size, which accords with the almost independent association between spatial distances and genetic differences suggested by the low correlations of Table 3. It thus appears that under the marginal conditions, which are set by the spatial distribution of the individuals and by their genotypes in the four stands, both descriptors of spatiogenetic covariation detect no significant differences between the stands despite the conceptual distinctness of the descriptors. This suggests that, if there are any structural differences at all between stands, they are likely to be found in descriptors relating more directly to spatiogenetic clustering characteristics (such as the average degree of spatiogenetic separation between individuals and its standard deviation considered in the next section) and, ultimately in the spatiogenetic clustering patterns themselves.

**Table 4:** Observed average asymmetries between spatial distance and genetic difference (in spatial units), and their effective ranges of potential variation for three measures of genetic difference

stand	*d*_0_		*d*_*J*_		*d*_*ur*_	
	a¯sg MathType@MTEF@5@5@+=feaafiart1ev1aaatCvAUfKttLearuWrP9MDH5MBPbIqV92AaeXatLxBI9gBaebbnrfifHhDYfgasaacH8akY=wiFfYdH8Gipec8Eeeu0xXdbba9frFj0=OqFfea0dXdd9vqai=hGuQ8kuc9pgc9s8qqaq=dirpe0xb9q8qiLsFr0=vr0=vr0dc8meaabaqaciGacaGaaeqabaqabeGadaaakeaacuWGHbqygaqeamaaBaaaleaacqWGZbWCcqWGNbWzaeqaaaaa@3103@	*p*	a¯sg MathType@MTEF@5@5@+=feaafiart1ev1aaatCvAUfKttLearuWrP9MDH5MBPbIqV92AaeXatLxBI9gBaebbnrfifHhDYfgasaacH8akY=wiFfYdH8Gipec8Eeeu0xXdbba9frFj0=OqFfea0dXdd9vqai=hGuQ8kuc9pgc9s8qqaq=dirpe0xb9q8qiLsFr0=vr0=vr0dc8meaabaqaciGacaGaaeqabaqabeGadaaakeaacuWGHbqygaqeamaaBaaaleaacqWGZbWCcqWGNbWzaeqaaaaa@3103@	*p*	a¯sg MathType@MTEF@5@5@+=feaafiart1ev1aaatCvAUfKttLearuWrP9MDH5MBPbIqV92AaeXatLxBI9gBaebbnrfifHhDYfgasaacH8akY=wiFfYdH8Gipec8Eeeu0xXdbba9frFj0=OqFfea0dXdd9vqai=hGuQ8kuc9pgc9s8qqaq=dirpe0xb9q8qiLsFr0=vr0=vr0dc8meaabaqaciGacaGaaeqabaqabeGadaaakeaacuWGHbqygaqeamaaBaaaleaacqWGZbWCcqWGNbWzaeqaaaaa@3103@	*p*
Laubach A	**.408 ** (.402; .414)	.53	**.389 ** (.382; .392)	.39	**.369 ** (.364; .376)	.62
Laubach C	**.389 ** (.382; .399)	.65	**.373 ** (.364; .379)	.44	**.353 ** (.348; .363)	.75
Horn	**.401 ** (.395; .407)	.55	**.382 ** (.370; .386)	.48	**.374 ** (.367; .379)	.48
Karlshafen	**.393 ** (.390; .402)	.80	**.376 ** (.374; .384)	.86	**.365 ** (.358; .370)	.50

a¯sg
 MathType@MTEF@5@5@+=feaafiart1ev1aaatCvAUfKttLearuWrP9MDH5MBPbIqV92AaeXatLxBI9gBaebbnrfifHhDYfgasaacH8akY=wiFfYdH8Gipec8Eeeu0xXdbba9frFj0=OqFfea0dXdd9vqai=hGuQ8kuc9pgc9s8qqaq=dirpe0xb9q8qiLsFr0=vr0=vr0dc8meaabaqaciGacaGaaeqabaqabeGadaaakeaacuWGHbqygaqeamaaBaaaleaacqWGZbWCcqWGNbWzaeqaaaaa@3103@ := average spatiogenetic asymmetry based on spatial units, *p *:= proportion of asymmetries among 10.000 permutations exceeding a¯sg
 MathType@MTEF@5@5@+=feaafiart1ev1aaatCvAUfKttLearuWrP9MDH5MBPbIqV92AaeXatLxBI9gBaebbnrfifHhDYfgasaacH8akY=wiFfYdH8Gipec8Eeeu0xXdbba9frFj0=OqFfea0dXdd9vqai=hGuQ8kuc9pgc9s8qqaq=dirpe0xb9q8qiLsFr0=vr0=vr0dc8meaabaqaciGacaGaaeqabaqabeGadaaakeaacuWGHbqygaqeamaaBaaaleaacqWGZbWCcqWGNbWzaeqaaaaa@3103@, second line in parentheses := lower and upper 0.05-quantile specifying the effective range of variation of spatiogenetic asymmetry obtained for 10.000 permutations

### Average and variance of spatiogenetic separation

Table 5 summarizes the results on spatiogenetic separation. In contrast with the previous findings on covariation it turns out that the descriptors of spatiogenetic separation vary distinctly among stands. For both the observed average degrees and the standard deviations of separation as well as for all three measures of genetic difference, the stands Laubach A and Laubach C are clearly distinguished from the stands Horn and Karlshafen with even non-overlapping effective ranges of potential variation for *d*_0 _and *d*_*ur*_. In these cases the effects of the spatiogenetic marginal conditions can be held to be primarily responsible for the distinction. With increasing overlap between effective ranges of potential variation, effects of distribution of genotypes over spatial positions (i.e. intrinsically structural effects) gain weight in bringing about the differences between stands. Average spatiogenetic separation is smallest across all stands for the measure *d*_0 _of genetic difference and distinctly larger for *d*_*ur*_, while it is intermediate between these two for the measure *d*_*J*_. This corresponds to the property of *d*_0 _to measure the minimum genetic difference and *d*_*ur *_to be positive for identical heterozygotes.

**Table 5:** Observed average and standard deviation of spatiogenetic separation of individuals, and their effective ranges of potential variation for four stands and three measures of genetic difference

	*d*_0_		*d*_*J*_		*d*_*ur*_		*d*_0_	
stand	*μ*_*sg *_[m]	*p*	*μ*_*sg *_[m]	*p*	*μ*_*sg *_[m]	*p*	*μ*_*sg *_[m]	*μ*_*sg*/*μs*_
	*σ*_*sg *_[m]	*p*	*σ*_*sg *_[m]	*p*	*σ*_*sg *_[m]	*p*	*σ*_*sg *_[m]	
Laubach A	**15.6 ** (15.2; 16.6)	.77	**16.4 ** (16.2; 17.6)	.88	**24.2 ** (23.5; 24.4)	.15	**4.0**	**3.9**
	**6.3 ** (5.6; 6.7)	.30	**6.3 ** (5.6; 6.7)	.27	**7.4 ** (7.3; 8.2)	.94	**2.1**	
Laubach C	**17.5 ** (17.5; 19.5)	.95	**18.8 ** (18.9; 20.9)	.97	**24.1 ** (24.0; 25.4)	.93	**6.9**	*2.5*
	**6.5 ** (4.9; 6.7)	.09	**6.2 ** (4.8; 6.5)	.13	**7.2 ** (6.4; 7.7)	.32	**2.8**	
Horn	**21.4 ** (21.9; 23.7)	.99	**23.1 ** (23.2; 25.1)	.96	**33.3 ** (33.0; 34.2)	.76	5.7	**3.8**
	**9.0 ** (8.2; 9.6)	.39	**8.8 ** (7.9; 9.3)	.33	**13.4 ** (12.5; 13.6)	.19	**2.3**	
Karlshafen	**23.7 ** (24.0; 26.1)	.99	**25.7 ** (25.3; 27.5)	.87	**35.6 ** (35.8; 37.2)	.98	**8.0**	**3.0**
	**8.6 ** (8.1; 9.7)	.70	**8.3 ** (8.0; 9.6)	.83	**12.4 ** (11.0; 14.0)	.33	**2.1**	

*μ*_*sg *_:= observed average spatiogenetic separation in spatial units; *σ*_*sg *_:= observed standard deviation of spatiogenetic separation; *p *:= proportion of averages and standard deviations among 10.000 permutations exceeding *μ*_*sg *_and *σ*_*sg*_, respectively; in parentheses:= lower and upper 0.05-quantile specifying the effective range of variation of the respective separation parameters obtained for 10.000 permutations; *μ*_*s *_:= observed average spatial separation; *σ*_*s *_:= observed standard deviation of spatial separation.

Moreover, with the exception of Laubach A, the observed average separations of all stands are close to their lower 0.05-quantiles (consistently high *p*-values for *d*_0_, less consistently high *p*-values for *d*_*J *_and *d*_*ur*_). The variances show no such extreme behavior with respect to the position of observed values in their effective ranges of potential variation. The consistently smaller effective ranges of potential variation of average separation for the genetic difference measure *d*_*ur *_suggests this measure to be the least sensitive to permutations of spatial position. This deviation from the other measures of genetic difference also concerns the position of the observed separation within its effective range of potential variation, which is closer to the upper 0.05-quantile within this range for Laubach A.

The ranking of the stands with respect to the average spatiogenetic separation is almost the same for all measures of genetic difference and this reflects their ranking according to intensity and type of thinning and of age of stands (see Table 2): the averages increase starting with weak low thinning (Laubach A) and continuing with low thinning (Laubach C), high thinning and intermediate average age (Horn), and high thinning and higher average age (Karlshafen). The same ranking results with respect to overall stand density, however with much more pronounced differences between the Laubach stands and smaller differences between the other two stands (see Table 2). Thus, the observed average spatiogenetic asymmetry within stands is apparently not large enough to genetically dissolve or even reverse the general tendencies of spatial clustering. This might indicate (but does not prove) a tendency for lower genetic differences within than between disjoint spatial clusters. The tendency is weakest in stands with artificial planting (Horn and Karlshafen, see Table 2), as was to be expected and as is confirmed by the distinct increase of the standard deviation of separation in these stands.

A more direct comparison between spatiogenetic separation and spatial distribution characteristics is obtained from computation of the purely spatial separations, i.e. the smallest spatial distance of an individual from all other individuals. The reference of spatiogenetic commensurability to the spatial component allows us to directly compare spatial with spatiogenetic separations. In particular, the minimum spatiogenetic difference of an individual from all other individuals always exceeds or is equal to its minimum spatial distance. If any of the spatially nearest neighbors has a genetic difference from the reference individual that is smaller than or equal to the spatial distance, then the minimum spatiogenetic difference equals the minimum spatial distance. Hence, if the minimum spatiogenetic difference properly exceeds the minimum spatial distance than the genetic differences of all spatially nearest neighbors properly exceed their spatial distance. This is true in our stands. As is shown in the next to the rightmost column of Table 5, average spatial separations are distinctly smaller than average spatiogenetic separations with ratios *μ*_*sg*_/*μ*_*s *_varying for *d*_0 _between 2.5 and 3.9 (rightmost column). As opposed to the last suggestion, this can be taken as an indication for a tendency of genetic differences to dissolve the patterns of spatial clustering at the levels of nearest neighborhood. This, however, need not extend to higher levels of hierarchy at which clusters contain disjoint subclusters.

Table 5 also shows that the ranking of stands changes with respect to their average spatial separations and thus the stand densities (the order of Laubach C and Horn is reversed), and that the average spatial separation varies more strongly among stands than does average spatiogenetic separation (*μ*_*s *_in Karlshafen doubles that in Laubach A). Reversals of ranking with respect to stand density and average spatial separation can be explained by stronger spatial structuring (clustering, fragmentation) in the stand of lower density (Horn). The higher average spatiogenetic separation together with the distinctly higher *μ*_*sg*_/*μ*_*s *_ratio in Horn as compared to Laubach C in turn hints at a stronger tendency in Horn to dissolve spatial structures through a more equal distribution of genotypes over spatial clusters of relatively high density. The above observations and their apparently controversial assessments depend on associations between genotypes and locations to the degree that these can vary under the marginal restrictions provided by the distribution of individuals in space and by the kinds and frequencies of genotypes. Because the potential variation of the average asymmetries, for example, is very small and does not allow for values close to zero (as demonstrated in Table 4), the marginal restrictions *a priori *prohibit situations of strong spatiogenetic clustering, where spatially separated groups of individuals are genetically clearly distinguished from other such groups. Another effect adding to this restriction could be found in the fact that genetic differences for the studied gene loci show a distinctly lower resolution than spatial distances. Under these restrictions, the observation that the average spatiogenetic separations are, with a few exceptions, realized very close to their lower 0.05-quantile deserves further consideration.

In fact, within the limits set by the marginal restrictions and the resolution of the genetic differences, all stands can be considered to show very low spatiogenetic separation. This lends more substantiated support to one of the above suggestions, namely that genetically similar individuals tend to gather in spatial clusters rather than to disperse over these clusters. It also confirms the above-mentioned ranking of the stands for their averages of spatiogenetic separation. The medium sized standard deviations hint at the possibility of variability of spatiogenetic differences within and between clusters. These considerations are of course only relevant if disjoint spatiogenetic clusters exist, which are sufficiently isolated. The pertaining findings will be presented in the next section.

### Spatiogenetic cluster structure

**Figure 1:**
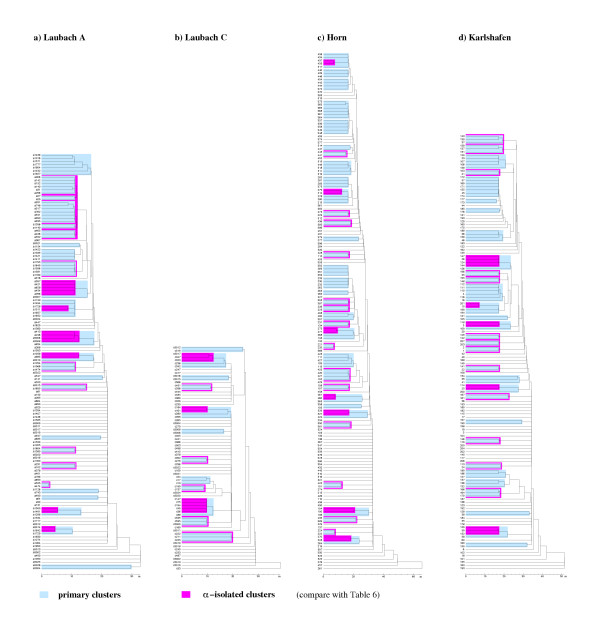
Spatiogenetic dendrograms of four beech stands based on *d*_0_.

The results obtained for the four elementary descriptors of cluster structure are summarized in Table 6. For purposes of illustration, spatiogenetic dendrograms based on the measure *d*_0 _of genetic difference are provided in Figure 1 for the four study stands. The two types of cluster structure, primary and *α*-isolated, for which the descriptors are calculated are highlighted in this figure. Averaging degrees of isolation over the primary clusters in the four stands (as explained above) results in *α *= 0.185 as the reference level of isolation based on the genetic difference *d*_0_. Likewise *α *= 0.159 and *α *= 0.153 for the reference levels based on *d*_*J *_and *d*_*ur*_, respectively.

**Table 6:** Four descriptors of cluster structure (poc := proportion of individuals organized in clusters, ecs := effective cluster size, acs := average cluster size, enc := effective number of clusters) for the primary and *α*-isolated cluster structures

**genetic difference ***d*_0 _**used in ***d*_*sg*_, *α *= 0. 185								
stand	primary cluster structure				*α*-isolated cluster structure			
	*poc*	*ecs*	*acs*	*enc*	*poc*	*ecs*	*acs*	*enc*

Laubach A	.644	4.29	3.40	19.79	.386	9.86	5.10	5.17
Laubach C	.507	3.56	3.00	10.13	.310	3.09	2.75	7.12
Horn	.652	3.54	3.06	30.21	.268	2.18	2.10	20.17
Karlshafen	.641	3.51	2.94	25.96	.296	2.48	2.33	16.96

**genetic difference ***d*_*J *_**used in ***d*_*sg*_, *α *= 0. 159								

stand	primary cluster structure				*α*-isolated cluster structure			
	*poc*	*ecs*	*acs*	*enc*	*poc*	*ecs*	*acs*	*enc*

Laubach A	.652	3.42	2.97	25.16	.295	2.44	2.29	16.01
Laubach C	.704	3.72	3.13	13.44	.394	2.43	2.33	11.53
Horn	.616	3.14	2.73	32.18	.244	2.00	2.00	20.00
Karlshafen	.592	3.07	2.63	27.35	.331	2.96	2.47	15.89

**genetic difference ***d*_*ur *_**used in ***d*_*sg*_, *α *= 0. 153								

stand	primary cluster structure				*α*-isolated cluster structure			
	*poc*	*ecs*	*acs*	*enc*	*poc*	*ecs*	*acs*	*enc*

Laubach A	.182	2.83	2.67	8.47	.045	2.00	2.00	3.00
Laubach C	.479	3.94	3.40	8.63	.296	2.71	2.63	7.74
Horn	.232	3.00	2.71	12.67	.146	2.25	2.18	10.67
Karlshafen	.296	5.14	3.23	8.17	.155	2.73	2.44	8.07

*N *:= number of individuals in a stand, *N*_*c *_:= number of individuals organized in clusters, *k *:= number of clusters, *n*_*i *_:= size of the _*i*_-th cluster. *poc *= *N*_*c*_/*N*, ecs=∑ini2/Nc
 MathType@MTEF@5@5@+=feaafiart1ev1aaatCvAUfKttLearuWrP9MDH5MBPbIqV92AaeXatLxBI9gBaebbnrfifHhDYfgasaacH8akY=wiFfYdH8Gipec8Eeeu0xXdbba9frFj0=OqFfea0dXdd9vqai=hGuQ8kuc9pgc9s8qqaq=dirpe0xb9q8qiLsFr0=vr0=vr0dc8meaabaqaciGacaGaaeqabaqabeGadaaakeaacqWGLbqzcqWGJbWycqWGZbWCcqGH9aqpdaaeqaqaaiabd6gaUnaaDaaaleaacqWGPbqAaeaacqaIYaGmaaGccqGGVaWlcqWGobGtdaWgaaWcbaGaem4yamgabeaaaeaacqWGPbqAaeqaniabggHiLdaaaa@3C67@, *acs *= *N*_*c*_/*k*, enc=Nc2/∑ini2
 MathType@MTEF@5@5@+=feaafiart1ev1aaatCvAUfKttLearuWrP9MDH5MBPbIqV92AaeXatLxBI9gBaebbnrfifHhDYfgasaacH8akY=wiFfYdH8Gipec8Eeeu0xXdbba9frFj0=OqFfea0dXdd9vqai=hGuQ8kuc9pgc9s8qqaq=dirpe0xb9q8qiLsFr0=vr0=vr0dc8meaabaqaciGacaGaaeqabaqabeGadaaakeaacqWGLbqzcqWGUbGBcqWGJbWycqGH9aqpcqWGobGtdaqhaaWcbaGaem4yamgabaGaeGOmaidaaOGaei4la8YaaabeaeaacqWGUbGBdaqhaaWcbaGaemyAaKgabaGaeGOmaidaaaqaaiabdMgaPbqab0GaeyyeIuoaaaa@3D50@; hence, *N*_*c *_= *poc·N *= *acs·k *= *ecs·enc*

The probably most conspicuous result consists in the distinct effects of the three measures of genetic difference on the descriptors of cluster structure. For example, based on measures *d*_0 _and *d*_*J *_of genetic difference, the proportion of individuals organized in primary clusters exceeds 50% in all stands, and, when based on the measure *d*_*ur*_, it is below 50% in all stands (with a minimum of only 18.2%). For individuals organized in *α*-isolated clusters, the proportions based on *d*_0 _and *d*_*J *_are also consistently larger than the proportions based on *d*_*ur*_.

There are also effects, which are consistent for all three measures of genetic difference: (*i*) across stands the proportion of individuals organized in *α*-isolated clusters is distinctly smaller and in several cases even less than half of the proportion organized in primary clusters; (*ii*) the stand of Horn shows the largest effective number of clusters for both cluster structures; (*iii*) with one exception (Laubach A based on *d*_0_) all four descriptors of cluster structure show higher values in the primary than in the *α*-isolated cluster structure; (*iv*) for two of the genetic differences, *d*_0 _and *d*_*J*_, the effective number of clusters in the primary cluster structure varies across stands in parallel with stand sizes. The fact that in all stands a higher proportion of individuals is organized in primary than in *α*-isolated cluster structures may be accepted as an explanation of finding (*iii*). However, there is no fixed mathematical relation among all descriptors (see the equations stated at the bottom of Table 6) as is demonstrated by the exception in the stand Laubach A based on *d*_0_. Looking for consistency in ranking of stands for their descriptors of cluster structure it turns out that this holds in only one case, namely in the *α*-isolated cluster structure based on *d*_0_. In fact, the four stands are consistently ranked by all four descriptors of cluster structure, with the effective number of clusters exactly inverting the order specified by the other descriptors. The ranking is Laubach A, Laubach C, Karlshafen and Horn, where Laubach A shows the by far largest proportion of individuals organized in clusters, effective and average cluster size (9.86 and 5.10 individuals), and the smallest effective number of clusters (5.17). This ranking does not follow the previously considered rankings for stand size, stand density and average spatiogenetic separation.

## Discussion

It is probably widely ignored that descriptors of covariation between genetic differences and spatial distances may depend to a considerable degree on the spatiogenetic marginal conditions specified by the spatial positions of the individuals and their genotypes. This dependence may imply a strong *a priori *restriction to the potential variation of the descriptors. To assess this phenomenon, permutation analyses are required to include the lower and upper *ε*-quantiles realized over permutations in addition to *p*-values. In our study the effective ranges of variation are consistently small, which indicates that effects of the marginal conditions may be so dominant that effects of the distribution of genotypes over spatial positions play an almost negligible role in the determination of descriptors of covariation. This situation makes it difficult to evaluate the significance of the observations in terms of intrinsically structural aspects.

The effects of the spatiogenetic marginal conditions are less dominant for the descriptors of spatiogenetic separation. In fact, the intrinsically structural forces seem to act in ways driving the average spatiogenetic separation in all stands towards the minima that are realizable under the respective marginal conditions. This tendency is less consistent for the measure *d*_*ur *_of genetic difference, where, however, this measure turned out to be the least discriminative among the three measures of genetic difference taken into consideration. Minimization of spatiogenetic separation requires forces which promote the formation of spatiogenetic clusters. Such forces are typically active through the quite limited effective ranges of dispersal of the heavy barochorous seeds of beech followed by natural regeneration and thinning. Estimates of average seed dispersal distances in beech range between 17.7 m [7] and 21.6 m [16], which are surprisingly close to the average spatiogenetic separations found in our stands for the measure *d*_0 _of genetic difference. This phenomenon awaits further analysis.

The differences in average spatiogenetic separation between stands is closely associated with stand density and average spatial separation, as can be expected in the presence of spatiogenetic clusters. The distinct increase of the variance of spatiogenetic separation in the stands of Horn and Karlshafen goes directly along with the plantings practiced in parts of these stands and with the implied randomization of genotypes over spatial positions. The requirement of continuous local regeneration for autochthony (see Appendix) is thus not fully met by these two stands.

The above suggestions become more explicit in the analyses of spatiogenetic cluster structure. Two elementary types of cluster structure are considered: (a) primary cluster structure, in which clusters are the largest with no true substructure (no disjoint subclusters), and (b) *α*-isolated cluster structure, in which clusters are the smallest to show a degree of isolation that equals or exceeds a specified level *α*. Among these two types of structure the latter indicates the existence of (up to *α*) distinct subpopulation structure. The former specifies the entities within which no further subpopulation structure can be realized irrespective of their degrees of isolation. In order to capture the basic level of isolation realized in the primary cluster structure, *α *is chosen in accordance with the average degree of primary cluster isolation. Concerning the proportion of individuals organized in each cluster structure as well as the effective number of clusters and their effective and average sizes it turned out that the three measures of genetic difference under consideration lead to different assessments of structure in several respects. They however agree in attributing distinctly larger proportions of individuals to primary than to *α*-isolated cluster structures in all stands. Hence, above the level of primary clusters, there is not much true subpopulation structure (in essence less than a third of a stand is organized in *α*-isolated clusters). Moreover, the stand of Horn with its different age classes and admixture of planted trees shows the largest effective number of spatiogenetic clusters across both types of cluster structure and all measures of genetic difference. This accords with the assessment based on spatiogenetic separation, where a tendency in Horn to dissolve spatial structures through partial random distribution of genotypes is suggested. Thus, the existence of overlapping generations, which would have qualified Horn as a stand with higher degree of autochthony (see Appendix), may imply less distinct spatiogenetic structure at the stand level.

A ranking of the stands, which is consistent for all four descriptors of cluster structure exists only for the most discriminative measure of genetic difference (*d*_0_). In this ranking Laubach A is the stand with the largest proportion of members organized in *α*-isolated clusters, the by far largest effective and average cluster size, and the smallest effective number of clusters. Laubach A is also the stand that experienced the lowest degree of thinning, which apparently allowed for the maintenance of relatively few and large spatiogenetically isolated clusters within the first generation of natural regeneration. Weak low thinning largely mimics natural selection in that only competitively inferior trees are removed.

The other extreme is realized by Horn with the lowest proportion organized in *α*-isolated clusters and the smallest effective and average cluster size. Horn and Karlshafen were subject to the same type of forest management, namely high thinning, but Horn would have been assigned a higher degree of autochthony due to the existence of a second generation at the same location. High thinning interferes most strongly with the stand's natural competition regime by changing the local competition relations. The implied selection, if any, is likely to be spread more evenly over the stand than is true for low thinning. This might have dissolved some spatiogenetic clusters or lowered their separation so as to lead to smaller proportions organized in clusters and smaller cluster sizes. Apparently, and in contrast with Karlshafen as well as with the other two stands, the second generation of natural regeneration has contributed further to a more even distribution of genotypes over local clusters at the stand level with the above consequences for clustering, and this tendency might have been enhanced by planting.

The stands, Laubach C and Karlshafen, which are of intermediate ranking, were subject to heavy low thinning and high thinning without a second generation. As was mentioned above, low thinning generally follows more directly the natural local selection regimes than does high thinning, and this relation is also true to a lower degree between weak and heavy low thinning. This accords precisely with the ranking according to the four descriptors of cluster structure.

## Conclusion

In summary, natural regeneration in beech produces spatiogenetic cluster structure which becomes weaker with increasing intensity and selectivity of thinning but also with the presence of overlapping generations. This corresponds to observations of Epperson and Alvarez-Buylla [19] and Hamrick et al. [20] that natural thinning in tropical trees leads to a reduction of fine-scale genetic structure. This statement also applies to spatiogenetic cluster structure at the average level of isolation of primary cluster structures and to common stand dimensions. The surprisingly high correspondence between our descriptors of spatiogenetic structure and forest management types confirms the appropriateness of the applied measure of cluster isolation and of the criterion for the choice of *α*. However, since gene flow generally does not respect artificial stand limits, studies of the present kind have to be extended beyond these limits in order to test higher order structures of population delineation.

## Methods

The analysis of structure in the spatial distribution of genetic characters basically depends on appropriate measures of difference within each of the two components. While there is usually no problem with the spatial component, the genetic component can be characterized by various aspects affecting the measurement of difference between the genotypes of individuals. The application of conventional methods based on variances or correlations (such as coefficients of autocorrelation) or on ordination techniques (such as principal component analyses) is restricted to quantitative measures (codings) of genotypes (for reviews see e.g. [21] and the book of Epperson [22]). Also some of these measures are difficult to extend to multiple alleles and loci. The approach of Smouse and Peakall [23] takes a special position in this context as it rests on a coding of genotypes (including multiple loci) by vectors with each component specifying the dosage of an allele in a genotype (or individual). Differences between genotypes are then essentially measured by half the squared Euclidean distance between these vectors (similar to the squared distance of Rogers [24]) with possibly different weights for the vector components.

Even though not always realized in the technical literature, the definition of autocorrelation of Smouse and Peakall ([23], equation (15)) based on their allele dosage vectors summarizes and generalizes most of the currently applied methods of spatiogenetic autocorrelation. Closer inspection even reveals that this approach includes what is occasionally referred to as "coancestry" (see e.g. [25]) or "kinship coefficient" (see e.g. [26]). To those who find autocorrelations difficult to interpret (since they are not correlations in the strict sense) the paper of Smouse and Peakall [23] provides a useful formal relationship between the coefficient of autocorrelation and their genetic difference measure (equations (12) and (13)). There is of course still the problem that autocorrelations depend on the way in which spatial distances are broken into distance classes and how these decompositions of distance can be justified.

This leaves us with types of analyses that rely on measures of spatial distance and genetic difference. Herewith, spatial distance may be measured along suitable gradients or paths connecting individuals, or it may even be replaced by ecological distances, when site characteristics are considered to be more significant than spatial distances. Among such analyses one can basically distinguish methods based on *spatiogenetic distribution parameters *from methods of *spatiogenetic clustering *(or fragmentation).

The former are chiefly applied with the aim to detect overall relations between spatial distance and genetic difference with the help of special descriptors of covariation. Spatiogenetic cluster methods, on the other hand, try to specifically identify groups or clusters of individuals, which are spatially adjacent and genetically similar. These clusters can in turn be used to derive new parameters of the joint distribution of spatial distances and genetic differences, which describe important structural aspects, as will be demonstrated in the following.

### Spatiogenetic cluster methods

These methods are based on the application of clustering algorithms to distance measures, which combine spatial distances and genetic differences into one measure that fulfills the above requirement of jointly quantifying genetic similarity and spatial vicinity. This can only be achieved by bringing both the measures of genetic difference and spatial distance on the same scale, so that they are commensurable. Commensurability guarantees that sizes of genetic differences can be assessed in relation to the sizes of the spatial distances. The combination of the commensurable difference measures into a joint spatiogenetic measure *d*_*sg *_of difference between individuals ought to consider (1) the proportion to which the spatial and genetic component contributes, and (2) the degree to which both components differ in their contributions. In order to simplify interpretation, these contributions should add linearily. As was recently demonstrated [9], the measure

dsg=a⋅dg'+(1−a)⋅ds'+c⋅|dg'−ds'|
 MathType@MTEF@5@5@+=feaafiart1ev1aaatCvAUfKttLearuWrP9MDH5MBPbIqV92AaeXatLxBI9gBaebbnrfifHhDYfgasaacH8akY=wiFfYdH8Gipec8Eeeu0xXdbba9frFj0=OqFfea0dXdd9vqai=hGuQ8kuc9pgc9s8qqaq=dirpe0xb9q8qiLsFr0=vr0=vr0dc8meaabaqaciGacaGaaeqabaqabeGadaaakeaacqWGKbazdaWgaaWcbaGaem4CamNaem4zaCgabeaakiabg2da9iabdggaHjabgwSixlabdsgaKnaaDaaaleaacqWGNbWzaeaacqGGNaWjaaGccqGHRaWkcqGGOaakcqaIXaqmcqGHsislcqWGHbqycqGGPaqkcqGHflY1cqWGKbazdaqhaaWcbaGaem4CamhabaGaei4jaCcaaOGaey4kaSIaem4yamMaeyyXICTaeiiFaWNaemizaq2aa0baaSqaaiabdEgaNbqaaiabcEcaNaaakiabgkHiTiabdsgaKnaaDaaaleaacqWGZbWCaeaacqGGNaWjaaGccqGG8baFaaa@5508@

has the desired properties. In this expression, dg'
 MathType@MTEF@5@5@+=feaafiart1ev1aaatCvAUfKttLearuWrP9MDH5MBPbIqV92AaeXatLxBI9gBaebbnrfifHhDYfgasaacH8akY=wiFfYdH8Gipec8Eeeu0xXdbba9frFj0=OqFfea0dXdd9vqai=hGuQ8kuc9pgc9s8qqaq=dirpe0xb9q8qiLsFr0=vr0=vr0dc8meaabaqaciGacaGaaeqabaqabeGadaaakeaacqWGKbazdaqhaaWcbaGaem4zaCgabaGaei4jaCcaaaaa@3059@ and ds'
 MathType@MTEF@5@5@+=feaafiart1ev1aaatCvAUfKttLearuWrP9MDH5MBPbIqV92AaeXatLxBI9gBaebbnrfifHhDYfgasaacH8akY=wiFfYdH8Gipec8Eeeu0xXdbba9frFj0=OqFfea0dXdd9vqai=hGuQ8kuc9pgc9s8qqaq=dirpe0xb9q8qiLsFr0=vr0=vr0dc8meaabaqaciGacaGaaeqabaqabeGadaaakeaacqWGKbazdaqhaaWcbaGaem4CamhabaGaei4jaCcaaaaa@3071@ are the commensurable versions of the original measures *d*_*g *_and *d*_*s *_of genetic difference and spatial distance between individuals, and *a *and *c *are weighting parameters such that 0 ≤ *a *≤ 1 and 0 ≤ *c *≤ min{*a*, 1 – *a*}. In all of the following analyses the choice *a *= *c *= 0.5 is made, since it gives equal weight to the genetic and the spatial component, and since the asymmetry |dg'
 MathType@MTEF@5@5@+=feaafiart1ev1aaatCvAUfKttLearuWrP9MDH5MBPbIqV92AaeXatLxBI9gBaebbnrfifHhDYfgasaacH8akY=wiFfYdH8Gipec8Eeeu0xXdbba9frFj0=OqFfea0dXdd9vqai=hGuQ8kuc9pgc9s8qqaq=dirpe0xb9q8qiLsFr0=vr0=vr0dc8meaabaqaciGacaGaaeqabaqabeGadaaakeaacqWGKbazdaqhaaWcbaGaem4zaCgabaGaei4jaCcaaaaa@3059@ - ds'
 MathType@MTEF@5@5@+=feaafiart1ev1aaatCvAUfKttLearuWrP9MDH5MBPbIqV92AaeXatLxBI9gBaebbnrfifHhDYfgasaacH8akY=wiFfYdH8Gipec8Eeeu0xXdbba9frFj0=OqFfea0dXdd9vqai=hGuQ8kuc9pgc9s8qqaq=dirpe0xb9q8qiLsFr0=vr0=vr0dc8meaabaqaciGacaGaaeqabaqabeGadaaakeaacqWGKbazdaqhaaWcbaGaem4CamhabaGaei4jaCcaaaaa@3071@| in the components is fully accounted for. For this choice of parameters *d*_*sg *_simplifies to *d*_*sg *_= max {ds'
 MathType@MTEF@5@5@+=feaafiart1ev1aaatCvAUfKttLearuWrP9MDH5MBPbIqV92AaeXatLxBI9gBaebbnrfifHhDYfgasaacH8akY=wiFfYdH8Gipec8Eeeu0xXdbba9frFj0=OqFfea0dXdd9vqai=hGuQ8kuc9pgc9s8qqaq=dirpe0xb9q8qiLsFr0=vr0=vr0dc8meaabaqaciGacaGaaeqabaqabeGadaaakeaacqWGKbazdaqhaaWcbaGaem4CamhabaGaei4jaCcaaaaa@3071@, dg'
 MathType@MTEF@5@5@+=feaafiart1ev1aaatCvAUfKttLearuWrP9MDH5MBPbIqV92AaeXatLxBI9gBaebbnrfifHhDYfgasaacH8akY=wiFfYdH8Gipec8Eeeu0xXdbba9frFj0=OqFfea0dXdd9vqai=hGuQ8kuc9pgc9s8qqaq=dirpe0xb9q8qiLsFr0=vr0=vr0dc8meaabaqaciGacaGaaeqabaqabeGadaaakeaacqWGKbazdaqhaaWcbaGaem4zaCgabaGaei4jaCcaaaaa@3059@}.

Commensurability can be achieved in at least three ways: by division of *d*_*g *_and *d*_*s *_by their respective maxima, or by expressing the genetic component in terms of spatial units or vice versa. The latter case is realized by ds'
 MathType@MTEF@5@5@+=feaafiart1ev1aaatCvAUfKttLearuWrP9MDH5MBPbIqV92AaeXatLxBI9gBaebbnrfifHhDYfgasaacH8akY=wiFfYdH8Gipec8Eeeu0xXdbba9frFj0=OqFfea0dXdd9vqai=hGuQ8kuc9pgc9s8qqaq=dirpe0xb9q8qiLsFr0=vr0=vr0dc8meaabaqaciGacaGaaeqabaqabeGadaaakeaacqWGKbazdaqhaaWcbaGaem4CamhabaGaei4jaCcaaaaa@3071@ = *d*_*s *_and dg'
 MathType@MTEF@5@5@+=feaafiart1ev1aaatCvAUfKttLearuWrP9MDH5MBPbIqV92AaeXatLxBI9gBaebbnrfifHhDYfgasaacH8akY=wiFfYdH8Gipec8Eeeu0xXdbba9frFj0=OqFfea0dXdd9vqai=hGuQ8kuc9pgc9s8qqaq=dirpe0xb9q8qiLsFr0=vr0=vr0dc8meaabaqaciGacaGaaeqabaqabeGadaaakeaacqWGKbazdaqhaaWcbaGaem4zaCgabaGaei4jaCcaaaaa@3059@ = *x*·*d*_*g*_, where the normalization factor *x *comes from minimizing the sum ∑(*d*_*s *_- *x*·*d*_*g*_)^2 ^over all pairs of individuals. Analogously, spatial distances are expressed in terms of genetic units by dg'
 MathType@MTEF@5@5@+=feaafiart1ev1aaatCvAUfKttLearuWrP9MDH5MBPbIqV92AaeXatLxBI9gBaebbnrfifHhDYfgasaacH8akY=wiFfYdH8Gipec8Eeeu0xXdbba9frFj0=OqFfea0dXdd9vqai=hGuQ8kuc9pgc9s8qqaq=dirpe0xb9q8qiLsFr0=vr0=vr0dc8meaabaqaciGacaGaaeqabaqabeGadaaakeaacqWGKbazdaqhaaWcbaGaem4zaCgabaGaei4jaCcaaaaa@3059@ = *d*_*g *_and ds'
 MathType@MTEF@5@5@+=feaafiart1ev1aaatCvAUfKttLearuWrP9MDH5MBPbIqV92AaeXatLxBI9gBaebbnrfifHhDYfgasaacH8akY=wiFfYdH8Gipec8Eeeu0xXdbba9frFj0=OqFfea0dXdd9vqai=hGuQ8kuc9pgc9s8qqaq=dirpe0xb9q8qiLsFr0=vr0=vr0dc8meaabaqaciGacaGaaeqabaqabeGadaaakeaacqWGKbazdaqhaaWcbaGaem4CamhabaGaei4jaCcaaaaa@3071@ = *y*·*d*_*s*_, where *y *comes from minimizing ∑(*d*_*g *_- *y*·*d*_*s*_)^2 ^[9]. In the present paper preference will be given to commensurability of genetic differences with spatial distances. This scaling, in which genetic differences are specified in terms of spatial distances, better reflects our spatially determined concepts of population delineation. Clustering of individuals will be based on the single-linkage algorithm applied to *d*_*sg*_. This algorithm is preferred because of its high resolution, intuitive appeal and conceptual rigor (see [28,29]).

The results of clustering will be presented in the common form of dendrograms, in which each cluster (or group) appears as the "leaves" or tips of a branch. The thus obtained description of spatiogenetic cluster structure will provide the basis for an assessment of subpopulation structure and spatiogenetic coherence.

### Characterization and quantification of subpopulation structure

One way of describing the distinctness of subpopulation structure consists in determining degrees of spatiogenetic isolation of the groups. The measure of *cluster isolation *applied in the following goes back to an idea of Estabrook [27] and is detailed in two papers of Gregorius [28,29]. For *I*_*G *_as the internal differentiation of group *G *(largest difference among spatiogenetically nearest neighbors in the group) and *E*_*G *_as the external differentiation (smallest spatiogenetic difference between members of the group and individuals from outside the group), the measure takes the form (*E*_*G *_- *I*_*G*_)/*E*_*G*_. Spatiogenetic groups or clusters always satisfy *E*_*G *_> *I*_*G*_. Complete isolation, where (*E *- *I*)/*E *= 1, is realized only for solitary individuals, i.e. those individuals not belonging to any spatiogenetic group.

Basically, subpopulations are required to appear as disjoint spatiogenetic groups with distinct degrees of isolation. In general, any partition of a set of individuals into disjoint clusters with some individuals possibly remaining un-clustered will be referred to as a *cluster structure*. Such a structure can be addressed as "true" or "complete" according to whether it contains at least two clusters or consists solely of clusters (all individuals organized in clusters). These cluster structures can be further characterized by the number of clusters, their sizes and degrees of isolation, or by the proportion of members of the stand organized in clusters (indicating the completeness of the cluster structure). The pertaining measures will be referred to as *descriptors of cluster structure*. A common method of partitioning is known as "cutting stems" in dendrograms, and it consists of all clusters of maximal size not exceeding a specified internal differentiation (hierarchy level). This type of cluster structure will not be pursued further in this paper because of the difficulty to interpret its structural characteristics.

In fact, there may be no true cluster structure at all if no group exists except of the whole stand, which is typical of identical differences between all spatiogenetically nearest neighbors ("ties"). Another extreme structural feature arises, when no disjoint groups consisting of at least two individuals exist, so that the groups form a completely nested sequence (also known as complete "chaining"). This indicates the existence of a center around which spatiogenetic coherence gradually decreases. True cluster structure in the above sense of a partition of a stand into at least two disjoint groups does therefore again not exist. This concept of absence of (true) cluster structure can be projected down to the level of individual clusters in that the members of such a cluster form a chain or a tie. Such a cluster will be referred to as a *primary cluster *or group if it is the largest cluster containing no disjoint subclusters (recall that by definition subclusters consist of at least two individuals). Primary clusters may contain or even completely consist of individuals connected by ties. Since two primary clusters are either identical or disjoint, they establish a special partition of the stand. Among the individuals not belonging to a primary cluster are the solitary individuals. There may also exist individuals, which are neither solitary nor belong to a primary cluster. The cluster structure resulting from the partition of a stand into primary clusters and the remaining individuals is unique and does not depend on predefined hierarchy levels. It can therefore be viewed as an intrinsic structural characteristic. Since primary clusters are the largest clusters showing no true cluster substructure, it is justified to call the partition of a stand into its primary spatiogenetic clusters and the remaining individuals the *primary cluster structure*. Complete chaining as well as a complete tie constitute degenerate primary structures consisting of only one primary cluster each. The significance of the structure associated with each such partition is determined by the degrees of isolation of its constituent primary clusters.

The concept of primary structure can be consistently extended to higher order structures by considering each primary cluster as a single object (individual) in the otherwise unchanged clustering pattern. The definition of a primary cluster can then be applied to the thus reduced set of objects with its clustering pattern. This yields secondary clusters as the primary clusters in the reduced set of objects. These secondary clusters form a secondary cluster structure. Iterating this procedure leads to ever higher order structures until the whole stand is reduced to a single object. Higher order structures will however not be considered in this paper.

The idea of cluster isolation as introduced above gives rise to another basic type of cluster structure, which relates to the distinctness of cluster structure. With reference to a specified level *α*, say, of isolation (0 <*α *< 1), clusters can be distinguished, which show degrees of isolation equal to or greater than *α *and which contain no other cluster with at least this degree of isolation. Such a cluster will be called an *α*-*isolated cluster*, and it is the smallest cluster showing a degree of isolation ≥ *α*. Since, for a given level *α*, different clusters are disjoint, the totality of these clusters together with the remaining individuals again defines a cluster structure now referred to as *α*-*isolated cluster structure*.

In analogy with the primary cluster structure, an extension of the concept to higher order cluster structures can be obtained by considering each *α*-isolated cluster as a single object (individual) in the otherwise unchanged clustering pattern. A second order *α*-isolated cluster structure then results from application of the same principle of cluster formation to the thus reduced set of objects with its clustering pattern. As before, this procedure can be iterated to produce ever higher order structures until the whole stand is reduced to a single object. Again, in this paper the analyses will be restricted to the first order cluster structure.

Determination of levels a may depend on various criteria including a full account of *α*-isolated cluster structures for all admissible values of *α*. In the present paper, *α *will be referred to the primary cluster structures in the four stands studied, since these structures constitute the level below which no true substructure exists. For this purpose the weighted average of cluster isolation among all individuals organized in primary clusters will be taken for each stand (the weights are given by the cluster sizes). For these averages again the average over stands weighted by their sizes is taken in order to obtain a value for *α*, which is used likewise in all stands for determination of their *α*-isolated cluster structures.

Irrespective of its distinctness, any cluster structure can be characterized by the proportion of individuals organized in its clusters as was suggested above. This proportion equals 1 for complete cluster structures. Among the individuals organized in clusters, structural characteristics are basically determined by the number and the sizes of the constituent clusters. These two aspects can be combined in at least two ways into a single measure, one of which specifies cluster diversity in terms of the effective number of clusters and the other summarizes cluster sizes as their average. A third measure combines the former two measures to yield an effective cluster size through division of the number of individuals organized in clusters by the effective number of clusters. With *N*_*c *_:= number of individuals organized in clusters, *k *:= number of clusters, and *n*_*i *_:= size (number of individuals) of the *i*-th cluster (∑i=1kni=Nc
 MathType@MTEF@5@5@+=feaafiart1ev1aaatCvAUfKttLearuWrP9MDH5MBPbIqV92AaeXatLxBI9gBaebbnrfifHhDYfgasaacH8akY=wiFfYdH8Gipec8Eeeu0xXdbba9frFj0=OqFfea0dXdd9vqai=hGuQ8kuc9pgc9s8qqaq=dirpe0xb9q8qiLsFr0=vr0=vr0dc8meaabaqaciGacaGaaeqabaqabeGadaaakeaadaaeWaqaaiabd6gaUnaaBaaaleaacqWGPbqAaeqaaOGaeyypa0JaemOta40aaSbaaSqaaiabdogaJbqabaaabaGaemyAaKMaeyypa0JaeGymaedabaGaem4AaSganiabggHiLdaaaa@39F1@), the effective number of clusters (cluster diversity) is specified by the common index (∑i=1k(ni/Nc)2)−1=Nc2/∑i=1kni2
 MathType@MTEF@5@5@+=feaafiart1ev1aaatCvAUfKttLearuWrP9MDH5MBPbIqV92AaeXatLxBI9gBaebbnrfifHhDYfgasaacH8akY=wiFfYdH8Gipec8Eeeu0xXdbba9frFj0=OqFfea0dXdd9vqai=hGuQ8kuc9pgc9s8qqaq=dirpe0xb9q8qiLsFr0=vr0=vr0dc8meaabaqaciGacaGaaeqabaqabeGadaaakeaacqGGOaakdaaeWaqaaiabcIcaOiabd6gaUnaaBaaaleaacqWGPbqAaeqaaOGaei4la8IaemOta40aaSbaaSqaaiabdogaJbqabaGccqGGPaqkdaahaaWcbeqaaiabikdaYaaakiabcMcaPmaaCaaaleqabaGaeyOeI0IaeGymaedaaOGaeyypa0JaemOta40aa0baaSqaaiabdogaJbqaaiabikdaYaaakiabc+caVmaaqadabaGaemOBa42aa0baaSqaaiabdMgaPbqaaiabikdaYaaaaeaacqWGPbqAcqGH9aqpcqaIXaqmaeaacqWGRbWAa0GaeyyeIuoaaSqaaiabdMgaPjabg2da9iabigdaXaqaaiabdUgaRbqdcqGHris5aaaa@5096@, average cluster size equals *N*_*c*_/*k*, and effective cluster size equals ∑i=1kni2/Nc
 MathType@MTEF@5@5@+=feaafiart1ev1aaatCvAUfKttLearuWrP9MDH5MBPbIqV92AaeXatLxBI9gBaebbnrfifHhDYfgasaacH8akY=wiFfYdH8Gipec8Eeeu0xXdbba9frFj0=OqFfea0dXdd9vqai=hGuQ8kuc9pgc9s8qqaq=dirpe0xb9q8qiLsFr0=vr0=vr0dc8meaabaqaciGacaGaaeqabaqabeGadaaakeaadaaeWaqaaiabd6gaUnaaDaaaleaacqWGPbqAaeaacqaIYaGmaaGccqGGVaWlcqWGobGtdaWgaaWcbaGaem4yamgabeaaaeaacqWGPbqAcqGH9aqpcqaIXaqmaeaacqWGRbWAa0GaeyyeIuoaaaa@3AC4@. The product of the effective cluster size and the effective number of classes yields the number of individuals organized in clusters, as is required. These descriptors of cluster structure will be applied to characterize all cluster structures including primary and *α*-isolated.

Comparisons among stands for all of these structural features will then be performed in order to reveal characteristic differences in cluster structure among stands of different degrees of autochthony.

### Spatiogenetic distribution parameters

Among the most frequently applied descriptors of spatiogenetic structure are measures of covariation between genetic differences and spatial distances, which, in turn, are commonly specified in terms of the correlation coefficient. This coefficient will also serve as a reference in the present paper. The above measure *d*_*sg *_of spatiogenetic difference, however, provides further opportunities for exploration of variational aspects of structure. One of these is suggested by the average of the relative differences *a*_*sg *_:= |dg'
 MathType@MTEF@5@5@+=feaafiart1ev1aaatCvAUfKttLearuWrP9MDH5MBPbIqV92AaeXatLxBI9gBaebbnrfifHhDYfgasaacH8akY=wiFfYdH8Gipec8Eeeu0xXdbba9frFj0=OqFfea0dXdd9vqai=hGuQ8kuc9pgc9s8qqaq=dirpe0xb9q8qiLsFr0=vr0=vr0dc8meaabaqaciGacaGaaeqabaqabeGadaaakeaacqWGKbazdaqhaaWcbaGaem4zaCgabaGaei4jaCcaaaaa@3059@ - ds'
 MathType@MTEF@5@5@+=feaafiart1ev1aaatCvAUfKttLearuWrP9MDH5MBPbIqV92AaeXatLxBI9gBaebbnrfifHhDYfgasaacH8akY=wiFfYdH8Gipec8Eeeu0xXdbba9frFj0=OqFfea0dXdd9vqai=hGuQ8kuc9pgc9s8qqaq=dirpe0xb9q8qiLsFr0=vr0=vr0dc8meaabaqaciGacaGaaeqabaqabeGadaaakeaacqWGKbazdaqhaaWcbaGaem4CamhabaGaei4jaCcaaaaa@3071@|/max{ds'
 MathType@MTEF@5@5@+=feaafiart1ev1aaatCvAUfKttLearuWrP9MDH5MBPbIqV92AaeXatLxBI9gBaebbnrfifHhDYfgasaacH8akY=wiFfYdH8Gipec8Eeeu0xXdbba9frFj0=OqFfea0dXdd9vqai=hGuQ8kuc9pgc9s8qqaq=dirpe0xb9q8qiLsFr0=vr0=vr0dc8meaabaqaciGacaGaaeqabaqabeGadaaakeaacqWGKbazdaqhaaWcbaGaem4CamhabaGaei4jaCcaaaaa@3071@, dg'
 MathType@MTEF@5@5@+=feaafiart1ev1aaatCvAUfKttLearuWrP9MDH5MBPbIqV92AaeXatLxBI9gBaebbnrfifHhDYfgasaacH8akY=wiFfYdH8Gipec8Eeeu0xXdbba9frFj0=OqFfea0dXdd9vqai=hGuQ8kuc9pgc9s8qqaq=dirpe0xb9q8qiLsFr0=vr0=vr0dc8meaabaqaciGacaGaaeqabaqabeGadaaakeaacqWGKbazdaqhaaWcbaGaem4zaCgabaGaei4jaCcaaaaa@3059@}, which quantifies the average contribution of asymmetry to spatiogenetic differences. In some sense this *average spatiogenetic asymmetry *resembles the correlation, since complete symmetry is realized only for proportionality between spatial and genetic differences, and since this implies complete positive correlation. Similarly one expects for complete negative correlation large degrees of spatiogenetic asymmetry. Average asymmetry may therefore also be considered as a descriptor of spatiogenetic covariation.

Another, probably more basic characteristic of structure relates directly to the formation of spatiogenetic clusters. The single linkage method forms clusters according to nearest neighbor relationships, where the nearest neighbor of an individual is characterized by the smallest spatiogenetic difference of this individual from all other individuals. This difference will be called an individual's *degree of spatiogenetic separation*, and the frequency distribution of these degrees is seen to summarize the basic determinants of spatiogenetic clustering structure. The frequency distribution can be characterized in various ways including conventional statistical descriptors such as averages, variances or quantiles, all of which can be viewed as descriptors of spatiogenetic separation.

The average degree of separation and its standard deviation are descriptors of separation which can be related to subpopulation structure for the following reasons: (a) Small average degrees of separation imply small differences within spatiogenetic groups. Differences between disjoint groups may vary arbitrarily. Such situations are expected in stands with distinct family structures. However, the existence of solitary individuals (not belonging to any group) tends to increase the average. (b) Small variances in the degrees of separation indicate a spatiogenetic structure such that within each group, individuals tend to show about the same degrees of separation. Groups are therefore "flat" in the sense that they are close to ties, and the internal differentiation is similar for all groups. Again, this does not exclude the possibility of considerable variation in differences between disjoint groups. A potential cause for this kind of structure may be seen in a balance between cooperation and competition within limited distances among individuals. The balancing forces follow similar principles among groups with the result that the internal spatial distances and genetic differences are about the same for all groups.

The information from spatiogenetic cluster analyses and analyses of spatiogenetic distribution parameters will be combined in order to detect more pervasive aspects of cluster structure. For example, a stand may consist of a number of spatiogenetically well isolated groups (clusters) of individuals. If in addition a high spatiogenetic correlation would exist, this would indicate a consistent spatial arrangement of these groups in the sense that on the average spatially neighboring groups are genetically more similar than spatially distant groups. Otherwise, if the correlation is found to be low, such consistency does not exist possibly as the result of spatial irregularity of spatiogenetic cluster structure. This does however not affect the existence of distinct cluster structures.

### Assessment of spatiogenetic descriptors

Descriptors of structure usually indicate the presence and distinctness of specific structural characteristics. Any assessment of the distinctness of an observed descriptor value depends on the range of values that the descriptor can potentially take. For spatiogenetic structures this range is delineated by the number of individuals, their positions in space, and by the number and frequencies of genetic types realized in the collection of individuals. These conditions can be considered as setting the margins within which different assignments of individuals to spatial positions can realize different descriptor values. The ranges of potential variation and their bounds can only be explored by computing the descriptor values for all possible assignments of the individuals to their locations. Each assignment corresponds to a permutation of individuals over locations. In extreme cases, particularly if there is little up to no genetic variation, the descriptor may vary only negligibly among permutations. Thus, more realistic means of assessing the significance of the observed value are provided by considering the position of this value within the range of potential values in combination with the extension of this range and the proportion of assignments yielding equal or more extreme values of the descriptor than the observed value.

In fact, the latter proportion underlies what is commonly called a permutation test, and when applied to the correlation coefficient, it is known as the Mantel test. Sizes of potential ranges of the correlations are not considered in this test. As a test it is originally designed to reject the hypothesis of random distribution of genetic types over locations. This is a problematic concept, however, since small correlations can exist for erratic as well as highly regular (non-linear) structures, since any more or less regular spatiogenetic structure can come about by chance, and since the forces of structure formation are not explicitly addressed. We will therefore follow the above approach for the assessment of descriptors oriented at the extremeness of spatiogenetic structure characteristics. Extremeness is commonly measured by the *p*-value, i.e. the proportion of permutations yielding the observed or a larger (more extreme) descriptor value. Yet, if the range of potential values is small, the descriptor is revealed to be comparatively insensitive to permutations under the respective marginal conditions. Proportions of "extreme" values measured by the *p*-value are in this case not very informative. Otherwise, if the range of variation is sufficiently large and the *p*-value is small, it is justified to reject the hypothesis that the observed descriptor value is the result of random formation of structure. Herewith, random formation refers to the stipulation that all permutations are equally likely to be realized.

The number of individuals in the stands under investigation in the present paper is by far too large to allow realization of all permutations over locations. Therefore, Monte Carlo simulation will be applied to estimate the relevant quantities. In effect, such simulations yield random samples from the totality of all permutations. Under this restriction, the limits of the range of potential variation cannot be obtained with sufficient reliability. It is therefore more reasonable to refer to quantiles obtainable from the simulations, since these are less sensitive to the randomization. An "effective" range of potential variation would then be bounded from above by a threshold value that is reached or exceeded by a sufficiently small proportion of *ε *of all values (the upper *ε*-quantile). In the same way and for the same *ε *the range is bounded from below by its lower *ε*-quantile through the threshold value that is equal to or greater than a proportion *ε *of all values. This has the advantage of connecting ranges of potential variation to the notion of extremeness, where *ε *plays the role of a significance level.

### Measures of genetic difference

To provide for the possibility that spatiogenetic covariation and clustering may show up at different levels for distinct aspects of genetic resemblance among individuals, three common measures of genetic difference (specified in Table 1) are applied in all analyses. These measures are based on counts of individual genes the (allelic) states of which differ between two individuals or genotypes. It might be useful to recall that they are thus *genie difference measures*, which do not account for structural (e.g. location on the same chromosome) or functional (e.g. epistasis) aspects of a genotype. The minimum difference in (allelic) state between the individual genes of two individuals is measured by *d*_0_. The term "individual gene" is used here to address each gene individually irrespective of its state, so that for a diploid individual there are always 2·*l *individual genes at *l *gene loci. Individual genes can be homologous, in which case they are known as alleles, and two homologous genes may exhibit the same allelic state, in which case they are homoallelic. The degree of unrelatedness (*d*_*ur*_) corresponds directly to the complement of the well known coefficient of consanguinity (relatedness, kinship: the probability of drawing from two individuals two genes which are identical by descent or state). This is not to be confused with the coeffients used in the papers of Loiselle [25] and Kalisz et al. [26] under similar terms. These coefficients are not genetic difference measures between individuals nor coefficients of consanguinity but rather measures of autocorrelation, as was pointed out above. Making use of the identity between the coefficient of consanguinity of two individuals and the coefficient of inbreeding of any of their offspring, *d*_*ur *_can also be conceived of as measuring the genetic difference between two individuals by the expected relative degree of heterozygosity of their offspring. Note that *d*_*ur *_= 0 only for two genetically identical individuals if they are completely homozygous. The fact that *d*_*ur *_> 0 for genetically identical and heterozygous individuals shows that this measure does in parts not reflect common concepts of distance. Jaccard's index (*d*_*J*_), in turn, distinguishes individuals solely by gene states present in one and absent in the other individual. Additional copies of gene states found in homozygotes are not considered.

There are of course many other known or conceivable ways of measuring genetic differences between genotypes, even only among those based on counting and transforming gene differences. One of these is the measure of Smouse and Peakall [23], which was mentioned earlier. Leaving aside the transformation by squares and weights, these authors distinguish genotypes by the difference in number of copies of each allele. This is in fact identical to the principle underlying the measure *d*_0_, since the difference in number of copies of an allele is twice the number of replacements of alleles in one genotype to obtain the other. We decided in favour of *d*_0_, since the transformation applied by Smouse and Peakall [23] has as a counter-intuitive effect that genotypes sharing no alleles may show different measures (e.g. the measure between *A*_1_*A*_1 _and *A*_2_*A*_3 _equals 3, and that between *A*_1_*A*_1 _and *A*_2_*A*_2 _equals 4, while in both cases *d*_0 _= 1). Furthermore, since the measure of Smouse and Peakall [23] depends on number of alleles (and loci), it puts strong limitations on comparisons between populations and genetic markers without appropriate normalization.

## Appendix

The following compilation of criteria and characteristics of autochthony, which are considered in the present paper, are selected from the EU Council Directive 1999/105/EC [30] and other publications [31-34]. The basic criteria and characteristics refer to 1. continuity of local regeneration, 2. stand structure, 3. regularity of environment and their interrelations. Several indicators of these criteria are developed in the above publications, among which reproductive coherence and adaptational differentiation are of special relevance for the present study.

**1. ***Continuity of local regeneration *mainly depends on type and extent of regeneration.

**1.1 ***Type of regeneration *affects both genealogical continuity and adaptational processes. Two major types of regeneration can be distinguished, artificial and natural regeneration. While natural regeneration is mainly based on species-specific seed and pollen dispersal mechanisms, artificial regeneration includes seed translocation from external sources. This interrupts the local continuity of adaptational processes.

**1.1 ***Extent of natural regeneration *affects the adaptational capacity residing in an adult stand's genetic variation. The information available on this criterion was qualitative.

**2. ***Stand structure *affects adaptational differentiation on the levels of age class distribution and fragmentation.

**2.1 ***Age class distribution *is a result of an iteroparous reproduction mode, as is typical of forest trees. Continuous natural regeneration normally produces uneven-aged stands. Particular forest management types, however, may produce even-aged stands by clear cutting. Uneven-agedness indicates continuity of regenerative and adaptational processes and promotes the maintenance of genetic diversity.

**2.2 ***Fragmentation *at the level of stands influences the overall effective population size. Gene flow via pollen and seed preserves reproductive coherence of populations. Impairment of the mechanisms and operational conditions of gene flow may promote fragmentation and genetic differentiation. The studied stands were chosen because of the apparent lack of *a priori *fragmentation.

**3. ***Regularity of environmental conditions *enables populations to realize higher degrees of adaptedness possibly paid for by a loss of adaptability to unpredictable changes. Forest managment types can be considered as disturbances of the naturally regular environmental conditions.

**3.1 ***Forest management type *generally reduces stand density and, according to the intensity of thinning (forest management type), it influences mating systems. Therefore, forest management type affects effective population sizes of the species. Furthermore, reduced effective population sizes go along with losses of genetic variation and thus losses of adaptational capacity. Consequences of clear cutting and regeneration are mentioned above.

**3.2 ***Disturbance *(temporarily irregular impairment) may increase the genetic load carried by a population in order to preserve the adaptability to its regularly changing environmental conditions. Disturbance is a matter of concern in the studied stands only to the degree that it occurs through forest management.

## Authors' contributions

This paper is the result of intense cooperation between both authors on all topics. Both authors read and approved the final manuscript.
